# The association of biospheric values, environmental risk perception, and knowledge of environmental policies with pro-environmental behavioral intentions: the comparison across educational groups in China

**DOI:** 10.1186/s12889-026-28681-7

**Published:** 2026-07-29

**Authors:** Yun-Hsiang Tien, Jingchi Huang

**Affiliations:** School of Political Science and Public Administration, Wuhan University, Wuhan, 430072 China

**Keywords:** Biospheric values, Environmental risk perception, Knowledge of environmental policies, Pro-environmental behavioral intentions, China

## Abstract

Understanding the psychological processes that motivate individuals to engage in pro-environmental action is important. This study examines how three distinct psychological pathways—biospheric values (value-based motivation), environmental risk perception (affective risk appraisal), and knowledge of environmental policies (cognitive competence)—are associated with pro-environmental behavioral intentions, and how these relationships differ across educational attainment. Respondents were classified into less-educated, moderately educated, and highly educated groups, and hierarchical regression analyses were conducted. The results show that biospheric values and knowledge of environmental policies are significantly associated with pro-environmental intentions only among the less- and moderately educated groups. However, environmental risk perception is not significantly associated with intentions in any group. The psychological pathways leading to pro-environmental engagement are not uniform but differentiated by education. The study highlights the importance of tailoring environmental communication and education strategies to distinct motivational and cognitive profiles, offering theoretical and practical implications for promoting sustainable behavior in rapidly developing societies.

## Introduction

Environmental pollution has been a global problem that has attracted the attention of many governments worldwide [1]. In effectively responding to escalating environmental crises, there is a growing emphasis on individual pro-environmental behavior as a means to alleviate human impact on the natural world [2]. Pro-environmental behavioral intentions, which precede actual behavior, have been recognized as a critical precursor to meaningful environmental action [3]. Identifying the determinants of these intentions is undoubtedly important for promoting sustainable development, especially in rapidly industrializing nations such as China [4].

In recent years, pro-environmental behavior and its antecedents and outcomes have attracted attention and sparked discussions in academia [5]. Although numerous studies have explored the variables related to environmental awareness and actions, less is known about these related factors within socio-educational contexts. In China, given the impact of industrialization and urbanization on the environment, identifying the drivers of citizens’ pro-environmental intentions is undoubtedly of both theoretical and practical significance [6].

Biospheric values, which attributes intrinsic value to nature, has been shown to be associated with environmental protection [7]. Similarly, as a tangible threat, the perception of pollution may be related to protective behaviors, while knowledge of environmental policies equips individuals with the necessary knowledge to make meaningful decisions [8]. However, the extent to which these factors influence behavioral intentions and whether their effects vary across educational strata remains underexplored in the Chinese context.

From a psychological perspective, biospheric values reflects value-based moral motivation, environmental risk perception represents affective risk appraisal, and knowledge of environmental policies signifies cognitive competence. This study aims to address this gap by examining the roles of biospheric values, environmental risk perception, and knowledge of environmental policies in shaping pro-environmental behavioral intentions in China, with a specific focus on comparisons across different educational levels. This examination enables us to test distinct psychological mechanisms instead of merely considering the education level as a control variable.

The Chinese context provides a meaningful setting to examine these relationships. China has experienced rapid urbanization and industrialization over the past few decades, along with environmental deterioration and the establishment of a government-led environmental governance system [9]. Instead of emerging from civil society, China’s environmental governance is mainly characterized by top-down policy implementation, government-led public participation, and a gradually increasing citizens’ awareness of environmental protection [10]. This socio-political context may influence distinct psychological pathways by which individuals develop pro-environmental intentions.Consequently, examining how value-based, affective, and cognitive factors function in China, as well as whether these pathways differ across educational attainment, can clarify boundary conditions that confirm or challenge existing theories developed primarily in Western contexts. Utilizing nationally representative data in China, we seek to provide a deeper understanding of how educational attainment moderates intentions to engage in environmental protection. The findings are expected to inform educational and policy interventions aimed at enhancing public participation in environmental protection.

## Literature review and hypothesis

Pro-environmental behavior (PEB) refers to actions that minimize the negative impact on, and/or promote improvements to, the natural and built environment [11]. Pro-environmental behavioral intentions refer to actions aimed at being eco-friendly, focused on conserving natural resources, and enhancing environmental quality [12]. According to the Theory of Planned Behavior (TPB), intentions are theorized to precede behavior; however, intentions do not always guarantee behavior [13]. Some researchers analyzed the influence of pro-environmental intention on pro-environmental behavior, and most of them indicated that intentions were related to behaviors [14–16].

In the past, numerous researchers have investigated the correlation between pro-environmental behavioral intentions and many other variables [17, 18]. To provide a coherent theoretical base for this study, we use the Theory of Planned Behavior (TPB) as a general framework for understanding the psychological process from internal dispositions to behavioral intentions [19]. According to TPB, intentions are shaped by attitudes, subjective norms, and perceived behavioral control. In this study, we situate our variables within the broader value-attitude-behavior hierarchy, rather than merely mapping our three predictors directly onto these TPB constructs [20]. Specifically, biospheric values are conceptualized as a fundamental motivational goal that acts as a distal antecedent, guiding the formation of more specific attitudes toward environmental protection and corresponding pro-environmental actions [21]. Environmental risk perception refers to an affective-cognitive appraisal of threats. This process influences perceived vulnerability and the evaluation of potential outcomes, and it is closely related to attitudes. Knowledge of environmental policies enhances an individual’s perceived behavioral control by providing the necessary cognitive skills and helping them understand how to perform effective actions [22]. By organizing our variables with this hierarchical structure, we aim to clarify the distinct interconnected psychological pathways through which individuals form pro-environmental intentions.

These three predictors are interconnected in the process of shaping pro-environmental behavioral intentions. First, biospheric values provide the motivational foundation. From the perspective of cognitive consistency theory, individuals with strong ecocentric values are more likely to selectively attend to, perceive, and interpret environmental information that is congruent with their pre-existing value systems [23]. Therefore, they are more likely to perceive environmental risks and actively seek information that confirms their concerns. Second, the relationship between knowledge and values/perceptions is reciprocal. According to cognitive dissonance theory, when well-informed individuals recognize a discrepancy between their knowledge of environmental threats and their actual behavior, they may experience psychological discomfort [24]. This can motivate them to either strengthen their biospheric values or heighten their risk perception to justify or initiate behavioral change. Third, while risk perception can raise awareness, its transformation into concrete intentions often depends on the knowledge of feasible solutions. Thus, values motivate attention to risk, risk perception creates a demand for solutions, and knowledge helps translate concern into actionable intentions [25]. This integrated perspective is consistent with the TPB framework, where foundational values, affective threat appraisals, and cognitive competence jointly shape the motivation to act [26].

First, biospheric values refers to a nature-centered worldview that advocates for a shift in human actions to protect ecological systems and promote sustainability [27]. From the perspective of value-belief-norm (VBN) theory, biospheric values serve as an important antecedent of pro-environmental intentions by activating personal norms and a sense of moral obligation towards nature [28]. This perspective places the well-being of the entire ecological community above narrow and short-term human concerns [29]. Generally speaking, individuals with a strong ecocentric orientation are more likely to regard environmental protection as a moral imperative, and this orientation tends to co-occur with a sense of responsibility and a willingness to engage in pro-environmental actions [30]. Previous studies have revealed that ecocentric orientations are significantly associated with pro-environmental behaviors such as recycling, energy conservation, and support for environmental policies [31, 32]. Briefly, biospheric values is associated with individuals’ adoption of environmentally friendly practices. Consequently, based on the aforementioned discussion, we propose the following hypothesis:

- Hypothesis 1: Biospheric values is positively associated with pro-environmental behavioral intentions.

According to risk perception theory, we conceptualize environmental risk perception as a cognitive-affective appraisal of environmental threats [33]. The cognitive component encompasses individuals’ judgment of the seriousness of pollution, and the affective component involves feelings of worry and concern about possible consequences. Regarding the relationship between individuals’ perception of environmental problems and their subsequent actions, generally speaking, when people perceive pollution in their surroundings as a serious threat to their health and quality of life, it may be accompanied by a sense of concern and an obligation to take some actions [34]. This kind of perception is correlated with individuals’ actions to alleviate environmental damage or protect themselves from its influence [35]. In modern societies, problems such as air and water pollution are frequently mentioned in public discussions. Personal experience and awareness of pollution are likely to be associated with individuals’ devotion to environmental protection to mitigate the pollution [36]. Therefore, we propose the following hypothesis.

- Hypothesis 2: Environmental risk perception is positively associated with pro-environmental behavioral intentions.

Knowledge of environmental policies refers to knowledge of environmental concepts and issues, the attitudinal dispositions, motivation, cognitive abilities, and skills, as well as the confidence and appropriate behaviors to apply such knowledge in order to make effective decisions in a range of environmental contexts [37]. Not everyone knows the government’s environmental policies. Generally speaking, individuals who have a better understanding of environmental policies have a solid cognitive basis to recognize the importance of environmental protection and their own behaviors [38]. In other words, they may better understand how to align their personal actions with beneficial environmental outcomes, instead of being confused or unaware of what to do. Although knowledge itself may not guarantee actions, it is still an important factor that helps the formation of behavioral intentions. Consequently, we propose the following research hypothesis:

- Hypothesis 3: Knowledge of environmental policies is positively associated with pro-environmental behavioral intentions.

From an environmental psychology perspective, educational level is not only a demographic characteristic but also an important factor influencing individuals’ psychological pathways for engaging in environmental protection [39]. We ground our reasoning on two complementary theoretical perspectives. First, Inglehart’s post-materialism theory, though primarily focused on generational value shifts, provides an important insight [40]. Education is a primary mechanism that expands cognitive capabilities, exposure to diverse information, and analytical reasoning skills [41]. Second, from the perspective of cognitive resource theory, we posit that the relative importance of different psychological drivers varies across educational strata [42].

Although the Theory of Planned Behavior offers a framework for understanding how attitudes and perceived behavioral control shape intentions, it does not specify whether the importance of these antecedents differs across population subgroups. This is where the combined logic of post-materialism and cognitive resource theory becomes particularly valuable. While TPB explains the psychological mechanisms that shape intentions, these complementary perspectives help specify how educational attainment may differentiate the relative strength of these mechanisms across different population subgroups [43]. As societies become more economically secure and educational attainment expands, individuals tend to place greater emphasis on post-materialist or self-expression values rather than materialist concerns [44]. Education serves as a crucial mechanism in this value shift by expanding cognitive capabilities, exposure to diverse information, and analytical reasoning skills. Importantly, the theory implies the psychological drivers of environmental engagement may differ across educational levels [45]. When combined with the logic of the cognitive resource theory introduced above, this perspective suggests that higher education not only promotes pro-environmental values but also facilitates a shift from affective and value-based motivations toward more complex cognitive appraisals [46].

For less-educated individuals, the ability to access formal knowledge and policy information related to the environment is often limited [47]. Consequently, for this group, engagement in environmental protection may show a stronger association with moral and value-based motivations. Therefore, biospheric values, which reflects an intrinsic valuation of nature and moral responsibility toward the environment, may serve as a crucial motive.

Comparatively, for those with higher educational attainment, greater cognitive resources, information-processing capacity, and familiarity with environmental policies, their intentions to engage in environmental protection are more likely to relate to cognitive mechanisms such as knowledge of environmental policies and perceived behavioral efficacy rather than moral appeals [48]. In other words, this kind of individuals may need concrete knowledge and the belief that their actions may be associated with strong behavioral intentions [49]. Based on the above, this study suggests that educational attainment conditions different pathways in the formation of pro-environmental behavioral intentions. Therefore, we propose the following hypothesis:

- Hypothesis 4: The associations of biospheric values, environmental risk perception, and knowledge of environmental policies with pro-environmental behavioral intentions differ across educational attainment levels.

## Methods

### Research design

This study utilized data from the Chinese General Social Survey (CGSS), standing as the pioneering national, comprehensive, and ongoing academic survey initiative in China. It employs a multistage stratified sampling method, which is widely acknowledged in the academic community as the most effective approach for acquiring representative data with substantial scientific research value [50]. Within the CGSS, counties serve as the primary sampling units, followed by urban communities and rural villages as secondary sampling units, with households being randomly selected using a mapping sampling approach [51].

For the 2021 Chinese General Social Survey, 2741 participants responded to the environmental module of the survey. In this study, respondents were categorized into three educational level groups: the less-educated group (junior high school or below), the moderately educated group (senior high school or an associate’s degree), and the highly-educated group (bachelor’s degree or above).

All procedures performed in this study were in accordance with the ethical standards of the institutional research committee and with the 1964 Helsinki Declaration and its later amendments or comparable ethical standards. All participants were informed about the purpose and procedures of the study prior to their involvement. Informed consent was obtained from all individual participants included in the study.

### Measures

#### Pro-environmental behavioral intentions

To assess three kinds of pro-environmental behavioral intentions, four items were rated using a five-point Likert scale from “1 = very unwilling” to “5 = very willing”. This study referred to the Pro-Environmental Behavior Scale (PEBS) developed previously [52]. The items were as follows: “Regularly sort and dispose of household waste”, “Recycle and reuse household items”, “Be willing to discuss waste classification plans with other residents”, and “Be willing to volunteer regularly in public service activities to maintain environmental cleanliness”. Although the items describe concrete actions, respondents were asked to report their willingness to perform these behaviors, thereby capturing behavioral intentions rather than actual behavior. All item scores were coded such that a higher score indicated stronger pro-environmental behavioral intentions, and the items had good internal consistency (α = 0.80). The item scores were then averaged to present an overall pro-environmental behavioral intentions score.

#### Biospheric values

Biospheric values was assessed using four items designed to evaluate respondents’ general views on the relationship between human society and the environment. Due to the constraints of using secondary data from the CGSS, we adopted items that have been empirically validated to capture ecocentric attitudes toward nature [53]. These items were originally derived from the revised New Environmental Paradigm (NEP) scale. We acknowledge that in Stern’s VBN model, biospheric values are conceptualized as a broader motivational construct that precedes the NEP [54]. Following the New Ecological Paradigm tradition, items concerning human disruption of nature, the fragility of nature’s balance, and the possibility of ecological crisis can be used to indicate an ecological worldview or pro-environmental orientation. When interpreted as reflecting concern for nature beyond human utility, such items are also conceptually related to ecocentric environmental attitudes [55]. Participants were asked to indicate their level of agreement with the following statements on a five-point Likert scale ranging from 1 = “Strongly disagree” to 5 = “Strongly agree”: “The destruction of nature by human beings often leads to catastrophic consequences”, “Currently, human beings are abusing and damaging the environment”, “ The balance of nature is fragile and easily disrupted”, and “If everything continues as it is, we will soon face a serious environmental disaster”. All items were coded such that a higher score indicated a stronger ecocentric orientation. A composite score for biospheric values was calculated by averaging the scores of the four items. These four items were selected from the revised New Environmental Paradigm (NEP) scale, and they directly capture core ecocentric themes, including the harmful consequences of anthropocentrism, the abuse and damage humans cause to the environment, the fragility of nature’s balance, and the risk of inevitable disasters.The scale demonstrated acceptable internal consistency (α = 0.73), supporting its use as a composite measure.

#### Environmental risk perception

Environmental risk perception was measured by asking respondents to evaluate the severity of four main types of environmental issues in their local area. This study referred to previous studies and posed the question: “How serious do you think the following environmental problems are in the area where you live?” The four items were: air pollution, water pollution, soil pollution, and noise pollution [56]. Responses were recorded on a six-point Likert scale: 6 = “Very serious,” 5 = “Relatively serious,” 4 = “Moderate,” 3 = “Not too serious,” 2 = “Not serious,” and 1 = “This problem does not exist.” All items were coded so that a higher score indicated a stronger perception of pollution severity. The scale demonstrated good internal consistency (α = 0.85). A composite score for environmental risk perception was created by averaging the scores across the four items.

#### Knowledge of environmental policies

This study referred to the previously developed knowledge of environmental policies Scale for Adults (ELSA) and assessed knowledge of environmental policies by measuring respondents’ self-reported knowledge of four key environmental policies and initiatives in China [57]. Participants were asked: “How much do you know about the following environmental initiatives?” The four items were: The Air Pollution Prevention and Control Action Plan, The Water Pollution Prevention and Control Action Plan, The Soil Pollution Prevention and Control Action Plan, and The Public Participation Measures in Environmental Protection. Responses were captured on a four-point Likert scale: 1 = “Unaware”, 2 = “Know a little”, 3 = “Know some”, 4 = “Know well”. All items were coded such that a higher score indicated a higher level of knowledge of environmental policies. The scale demonstrated excellent internal consistency (α = 0.90). A composite score for knowledge of environmental policies was calculated by averaging the scores of the four items.

#### Educational level

Educational levels were divided into three groups: the less-educated group, the moderately educated group, and the highly-educated group, which signify junior high school or below, senior high school or an associate’s degree, and a bachelor’s degree or above, respectively.

#### Demographic characteristics

In this study, several demographic variables were incorporated: sex, age, partnership status, city living experience, employment status, religious belief, communist party membership, and annual household income. Sex was coded as 0 for females and 1 for males. Partnership status was assessed using a dummy variable: 0 for no and 1 for yes. To streamline response options and facilitate statistical analysis, “cohabitation,” “first marriage,” and “remarried” were coded as 1, whereas “unmarried,” “separated,” “divorced,” and “widowed” were coded as 0. Regarding the city living experience of the respondents, if they reported having lived in urban areas, it was coded as 1; if not, it was coded as 0. The study employed the question, “Did you work for more than one hour last week for earning money?” to gauge employment status, with “Yes” coded as 1 and “No” coded as 0. For religious belief, 0 indicated no belief and 1 indicated the presence of belief. Communist party membership was assessed by asking respondents about their political affiliation. If respondents reported being a member of the Communist Party of China, it was coded as 1; if not, it was coded as 0. The annual household income was calculated by taking the logarithm of the sum of the annual household income plus one.

### Statistical analysis

The experimental platform employed in this study was SPSS 27.0, developed by Beijing Cloud-wing Data Information Technology Co., Ltd., based in Beijing, China. Initially, descriptive statistics were computed to compare the three educational level groups. Subsequently, multiple regression models were formulated, with pro-environmental behavioral intentions as the dependent variable, and biospheric values, environmental risk perception, and knowledge of environmental policies as the independent variables. Statistical significance was set at *P* < .05 for all analyses. The hierarchical regression was theory-driven. Model 1 included only demographic control variables. Model 2 incorporated biospheric values, which represent value-based moral motivation. Model 3 added environmental risk perception, which represents experiential and threat appraisal. Model 4 added knowledge of environmental policies, which represents cognitive competence and perceived behavioral control. This order facilitates the examination of the unique and incremental contribution of each predictor to pro-environmental behavioral intentions after accounting for the influence of previous variables.

Since multiple regression models were constructed, the continuous variables were tested for their distribution. The results of skewness (ranging from − 0.68 to 1.96) and kurtosis (ranging from − 0.73 to 4.80) indicated that these variables were normally distributed, suitable for regression models. To prevent potential multicollinearity, variance inflation factors (VIF) were calculated. The VIF values were low, ranging between 1.02 and 2.10.

### Conceptual framework

**Fig. 1:**
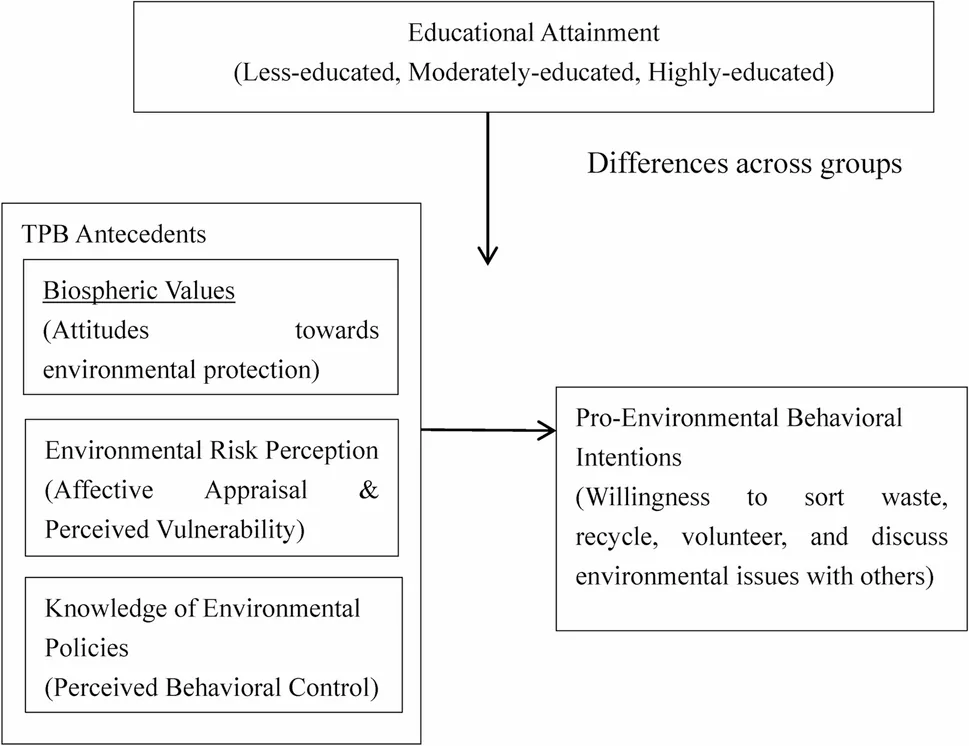
Conceptual framework

A conceptual framework is shown in Fig. 1. The framework is organized around three independent variables within the Theory of Planned Behavior (TPB). First, biospheric values capture individuals’ moral evaluation of environmental protection, reflecting the attitude component of TPB. Second, environmental risk perception represents the affective appraisal of pollution threats and perceived vulnerability, which may influence attitudes. Third, knowledge of environmental policies enhances individuals’ understanding of effective actions and how to execute them, thereby contributing to perceived behavioral control, a crucial antecedent of behavioral intentions in TPB.

In addition, the framework considers educational attainment as a grouping variable for comparison. According to Inglehart’s post-materialism theory, education differentiates the psychological pathways to pro-environmental intentions. For less-educated individuals, value-based moral motivations may be more relevant; for highly-educated individuals, cognitive mechanisms such as policy knowledge may play a stronger role. Therefore, the framework posits that the relationships between each independent variable and pro-environmental behavioral intentions may vary across educational attainment levels. 

## Results

### Demographic characteristics of participants

This study employed descriptive statistics to analyze the means and standard deviations of the measurement variables. Table 1 presents the statistical results for the demographic characteristics of respondents across three educational level groups. Significant differences were observed among the three age groups in all demographic variables. (Except for the *P*-value of religious belief, which is 0.002, all others are < 0.001.) For example, the proportion of males was highest in moderately educated group (52.1%), followed by the highly educated (47.5%) and less-educated groups (43.0%). The majority of respondents in the less-educated and moderately educated groups were in a relationship (79.3% and 66.6%, respectively), while a larger proportion of the highly educated group were single (55.4%). Religious belief was more prevalent in the less-educated group (7.8%) compared to the moderately educated (4.6%) and highly educated groups (4.1%). Employment rates increased with educational attainment, with 41.8% of the less-educated, 53.8% of the moderately educated, and 59.8% of the highly educated groups being employed. City living experience was more common among the highly educated (93.8%), followed by the moderately educated (80.4%) and the less-educated group (54.9%). Party membership also increased with educational level. 27.5% of the highly educated group reported party membership, compared to 17.2% of the moderately educated and 6.2% of the less-educated group. The less-educated group was significantly older (M = 58.31) than the moderately educated (M = 44.53) and highly educated groups (M = 33.43). Annual household income also increased with educational level, with the highly educated group reporting the highest income (M = 5.14), followed by the moderately educated (M = 4.82) and less-educated groups (M = 4.27).

**Table 1:** Demographic comparisons among three educational level groups

	Less-educated Group	Moderately- educated Group	Highly- educated Group	F or χ^2^	Sig.
	M (SD) or *N* (%)	M (SD) or *N* (%)	M (SD) or *N* (%)		
Sex				17.059	< 0.001
Male	720 (43.0)	374 (52.1)	163 (47.5)		
Female	954 (57.0)	344 (47.9)	180 (52.5)		
Relationship Status				181.135	< 0.001
Single	346 (20.7)	240 (33.4)	190 (55.4)		
Non-single	1328 (79.3)	478 (66.6)	153 (44.6)		
Religious Belief				12.408	0.002
Yes	131 (7.8)	33 (4.6)	14 (4.1)		
No	1543 (92.2)	685 (95.4)	329 (95.9)		
Employment Status				53.606	< 0.001
Yes	700 (41.8)	386 (53.8)	205 (59.8)		
No	974 (58.2)	332 (46.2)	138 (40.2)		
City Living Experience				836.028	< 0.001
Yes	2725 (54.9)	1697 (80.4)	984 (93.8)		
No	2242 (45.1)	414 (19.6)	65 (6.2)		
Communist Party Membership				455.671	< 0.001
Yes	308 (6.2)	363 (17.2)	288 (27.5)		
No	4659 (93.8)	1748 (82.8)	761 (72.5)		
Age	58.31 (14.48)	44.53 (17.21)	33.43 (13.46)	490.648	< 0.001
Annual Household Income	4.27 (1.16)	4.82 (0.77)	5.14 (0.42)	117.588	< 0.001

### Comparison of independent and dependent variables among three educational level groups

Table 2 shows the descriptive statistics for the core variables in our sample. The mean score for pro-environmental behavioral intentions was 3.94, with possible scores ranging from 1 to 5, indicating a relatively high level of willingness to engage in environmental protection. Biospheric values had a mean of 3.54, suggesting a relatively strong adherence to nature. Environmental risk perception had a mean of 3.15, above the midpoint of the 1–6 scale, reflecting a moderate level of perceived pollution severity. Knowledge of environmental policies had a relatively low mean of 1.27 on a 1–4 scale, indicating that, on average, respondents had limited familiarity with governmental environmental policies.

**Table 2:** Descriptive statistics of core variables

Variable	Mean	SD	Minimum	Maximum
Biospheric values	3.54	0.81	1	5
Environmental risk perception	3.15	1.18	1	6
Knowledge of environmental policies	1.27	0.45	1	4
Pro-environmental behavioral intentions	3.94	0.77	1	5

Statistical analysis revealed significant differences among the three educational groups across all key variables, as presented in Table 3. First, regarding biospheric values, the moderately educated group (M = 3.81) and the highly educated group (M = 3.81) reported significantly higher scores than the less-educated group (M = 3.34; *P* < .001). Post-hoc Bonferroni tests confirmed significant differences between the less-educated group and both the moderately educated and highly educated groups, while no significant difference was found between the two higher-educated groups. Second, for environmental risk perception, the highly educated group reported the highest mean score (M = 3.65), followed by the moderately educated group (M = 3.40), and the less-educated group (M = 2.93). The overall difference was statistically significant (*P* < .001), and pairwise comparisons indicated significant differences among all three groups. Third, regarding knowledge of environmental policies, the highly educated group demonstrated the highest level of literacy (M = 1.55), followed by the moderately educated group (M = 1.36), and the less-educated group (M = 1.18). The differences were statistically significant overall (*P* < .001), and Bonferroni tests confirmed significant differences between each pair of groups. Finally, for pro-environmental behavioral intentions, both the moderately educated (M = 4.04) and highly educated groups (M = 4.02) exhibited significantly stronger intentions compared to the less-educated group (M = 3.87; overall *P* < .001). Post-hoc analysis showed significant differences between the less-educated group and both the moderately educated and highly educated groups, while the two higher-educated groups did not differ significantly from each other.

**Table 3:** Mean comparison of core variables among three educational level groups

Variable	Less- educated Group	Moderately -educated Group	Highly- educated Group	F	Sig.	Bonferroni test
	M (SD)	M (SD)	M (SD)			
Biospheric values	3.34 (0.83)	3.81 (0.71)	3.81 (0.66)	102.779	< 0.001	(1,2) (1,3)
Environmental risk perception	2.93 (1.21)	3.40 (1.11)	3.65 (0.95)	78.766	< 0.001	(1,2) (1,3)(2,3)
Knowledge of environmental policies	1.18 (0.39)	1.36 (0.47)	1.55 (0.53)	122.539	< 0.001	(1,2) (1,3)(2,3)
Pro-environmental behavioral intentions	3.87 (0.81)	4.04 (0.70)	4.02 (0.66)	13.109	< 0.001	(1,2) (1,3)

(1) Less-educated group, (2) Moderately-educated group, (3) Highly-educated group

### Hierarchical regression analysis results by educational groups

Table 4 illustrates the impact of biospheric values, environmental risk perception, and knowledge of environmental policies on pro-environmental behavioral intentions. Model 1 included all control variables. From Model 2 to Model 4, biospheric values, environmental risk perception, and knowledge of environmental policies were successively added.

**Table 4:** Hierarchical regression model of factors associated with pro-environmental behavioral intentions among different educational level groups

Variable	Less-educated Group			
	Model 1	Model 2	Model 3	Model 4
	Beta(95% CI)	Beta(95% CI)	Beta(95% CI)	Beta(95% CI)
Sex (Ref: Female)	0.099** (0.054~0.251)	0.095** (0.047~0.245)	0.093** (0.045~0.243)	0.090** (0.040~0.237)
Relationship Status (Ref: Single)	0.020 (-0.083~0.162)	0.018 (-0.087~0.158)	0.018 (-0.087~0.157)	0.020 (-0.083~0.161)
Religious Belief (Ref: No)	− 0.028 (-0.266~0.101)	− 0.026 (-0.260~0.107)	− 0.026 (-0.260~0.107)	− 0.025 (-0.258~0.108)
Employment Status (Ref: No)	− 0.021 (-0.136~0.071)	− 0.020 (-0.135~0.072)	− 0.021 (-0.136~0.071)	− 0.027 (-0.145~0.063)
City Living Experience (Ref: No)	0.038 (-0.038~0.157)	0.030 (-0.051~0.144)	0.032 (-0.048~0.148)	0.028 (-0.055~0.141)
Communist Party Membership (Ref: No)	0.097** (0.090~0.433)	0.094** (0.083~0.425)	0.094** (0.081~0.424)	0.084** (0.055~0.400)
Age	− 0.093** (-0.009~-0.001)	− 0.085* (-0.008~-0.001)	− 0.087* (-0.008~-0.001)	− 0.084* (-0.008~-0.001)
Annual Household Income	0.056 (-0.005~0.0085)	0.052 (-0.008~0.082)	0.054 (-0.007~0.083)	0.052 (-0.008~0.081)
Biospheric values		0.072* (0.010~0.125)	0.075* (0.012~0.128)	0.074* (0.010~0.126)
Environmental risk perception			− 0.022 (-0.055 ~ 0.026)	− 0.025 (-0.057~0.024)
Knowledge of environmental policies				0.070* (0.014~0.233)
F	4.440***	4.544***	4.138***	4.218***
R^2^	0.034	0.039	0.040	0.044
∆F	4.440***	5.229*	0.501	4.859*
∆R^2^	0.034	0.005	0.000	0.005

Variable	Moderately-educated Group			
	Model 1	Model 2	Model 3	Model 4
	Beta (95% CI)	Beta (95% CI)	Beta (95% CI)	Beta (95% CI)
Sex (Ref: Female)	0.007 (-0.117~0.137)	0.008 (-0.113~0.136)	0.010 (-0.111~0.138)	0.000 (-0.124~0.125)
Relationship Status (Ref: Single)	0.128** (0.049~0.334)	0.124* (0.044~0.325)	0.122* (0.042~0.323)	0.126** (0.048~0.327)
Religious Belief (Ref: No)	− 0.042 (-0.429~0.150)	− 0.036 (-0.402~0.167)	− 0.035 (-0.402~0.168)	− 0.034 (-0.394~0.171)
Employment Status (Ref: No)	0.002 (-0.129~0.135)	− 0.022 (-0.162~0.100)	− 0.021 (-0.160~0.103)	− 0.023 (-0.163~0.098)
City Living Experience (Ref: No)	0.006 (-0.146~0.169)	− 0.018 (-0.189~0.124)	− 0.014 (-0.182~0.134)	− 0.009 (-0.172~0.142)
Communist Party Membership (Ref: No)	0.131** (0.058~0.393)	0.131** (0.060~0.390)	0.130** (0.060~0.389)	0.113* (0.029~0.360)
Age	0.006 (-0.004~0.005)	0.030 (-0.003~0.006)	0.023 (-0.003~0.005)	0.029 (-0.003~0.006)
Annual Household Income	0.132** (0.038~0.204)	0.129** (0.037~0.200)	0.129** (0.038~0.201)	0.121** (0.030~0.192)
Biospheric values		0.184*** (0.089~0.251)	0.187*** (0.091~0.254)	0.182*** (0.087~0.249)
Environmental risk perception			− 0.032 (-0.076~0.036)	− 0.039 (-0.079~0.032)
Knowledge of environmental policies				0.124** (0.051~0.292)
F	4.016***	5.573***	5.060***	5.381***
R^2^	0.063	0.095	0.096	0.110
∆F	4.016***	16.967***	0.496	7.860**
∆R^2^	0.063	0.032	0.001	0.015

Variable	Highly-educated Group			
	Model 1	Model 2	Model 3	Model 4
	Beta (95% CI)	Beta (95% CI)	Beta (95% CI)	Beta (95% CI)
Sex (Ref: Female)	− 0.072 (-0.262~0.071)	− 0.067 (-0.256~0.077)	− 0.067 (-0.254~0.077)	− 0.080 (-0.272~0.059)
Relationship Status (Ref: Single)	0.203* (0.036~0.502)	0.192* (0.021~0.488)	0.202* (0.035~0.500)	0.216* (0.054~0.518)
Religious Belief (Ref: No)	0.077 (-0.170~0.720)	0.065 (-0.216~0.678)	0.048 (-0.279~0.622)	0.047 (-0.280~0.616)
Employment Status (Ref: No)	− 0.134 (-0.382~0.010)	− 0.141* (-0.392~0.000)	− 0.128 (-0.374~0.017)	− 0.130 (-0.376~0.014)
City Living Experience (Ref: No)	0.038 (-0.361~0.645)	0.045 (-0.333~0.673)	0.049 (-0.317~0.685)	0.060 (-0.273~0.727)
Communist Party Membership (Ref: No)	0.108 (-0.036~0.349)	0.119 (-0.021~0.365)	0.115 (-0.025~0.359)	0.094 (-0.057~0.330)
Age	− 0.073 (-0.013~0.005)	− 0.082 (-0.013~0.005)	− 0.112 (-0.015~0.003)	− 0.124 (-0.016~0.003)
Annual Household Income	0.083 (-0.088~0.350)	0.076 (-0.099~0.339)	0.079 (-0.093~0.343)	0.075 (-0.099~0.335)
Biospheric values		0.099 (-0.028~0.229)	0.114 (-0.013~0.245)	0.108 (-0.019~0.238)
Environmental risk perception			− 0.113 (-0.170~0.011)	− 0.094 (-0.158~0.024)
Knowledge of environmental policies				0.126 (-0.002~0.308)
F	2.098*	2.142*	2.243*	2.408*
R^2^	0.065	0.074	0.086	0.100
∆F	2.098*	2.392	2.992	3.802
∆R^2^	0.065	0.009	0.011	0.014

p*<0.05; p**<0.01; p***<0.001

For the less-educated group, Model 1 revealed that being male (β = 0.099, *P* = .003), younger age (β = − 0.093, *P* = .008), and being a member of the Communist Party (β = 0.097, *P* = .003) were significantly associated with stronger pro-environmental behavioral intentions. When biospheric values was added in Model 2, it showed a significant positive association (β = 0.072, *P* = .022). This indicates that value-based motivation has an influential impact on the formation of intentions among citizens with relatively limited formal education. In Model 3, environmental risk perception was introduced but did not have a significant effect (β = − 0.022, *P* = .479). Finally, Model 4 incorporated knowledge of environmental policies, which was significantly positively associated with behavioral intentions (β = 0.070, *P* = .028), indicating that cognitive competence is also positively associated with intention formation within this group.

Among the moderately educated group, Model 1 revealed that being in a relationship (β = 0.128, *P* = .009), being a member of Communist Party (β = 0.131, *P* = .008), and having a higher annual household income (β = 0.132, *P* = .008) were significantly associated with pro-environmental behavioral intentions. After introducing biospheric values, Model 2 indicated that it had a strong and positive association (β = 0.184, *P* < .001), suggesting that moral and value-based orientations appear highly evident at middle levels of education. Model 3 added environmental risk perception, which was not significant (β = − 0.032, *P* = .482). In the final Model 4, knowledge of environmental policies showed a significant positive association (β = 0.124, *P* = .005), suggesting the common relevance of value-based and cognitive pathways for this group.

Regarding the highly educated group, Model 1 revealed that being in a relationship was the only demographic variable significantly associated with stronger pro-environmental behavioral intentions (β = 0.203, *P* = .024). When biospheric values was added to Model 2, its association was not significant (β = 0.099, *P* = .123), indicating that value-based motivation alone may not be sufficient to shape intentions at higher educational levels. Model 3 introduced environmental risk perception, which was also not significant (β = − 0.113, *P* = .085). In the final Model 4, knowledge of environmental policies was added, but it did not reach statistical significance (β = 0.126, *P* = .052). This indicates that cognitive mechanisms such as knowledge and perceived efficacy are not associated with the intentions of highly educated citizens.

To formally test whether the relationships differ across educational groups, a pooled regression analysis was conducted using the full sample. Dummy variables were created for educational groups, with the less-educated group serving as the reference category. Interaction terms were constructed by multiplying each dummy variable with the three core predictors. All continuous predictors were mean-centered prior to the creation of interaction terms to reduce multicollinearity. Three hierarchical models were estimated: Model 1 included only demographic control variables. Model 2 added the main effects of the three predictors and the educational dummies. Model3 added the six interaction terms. A significant interaction term indicates that the relationship between a predictor and pro-environmental behavioral intentions differs significantly between the specified educational group and the reference group. The results are presented in Table 5.

**Table 5:** Interaction analysis of educational attainment on the relationship between predictors and pro-environmental behavioral intentions

Variable	Model 1 (Control)	Model 2 (Main effects)	Model 3 (Interaction effects)
	Beta (95% CI)	Beta (95% CI)	Beta (95% CI)
Control variables			
Sex (Ref: Female)	0.055* (0.011~0.152)	0.042 (−0.009~0.131)	0.043 (−0.007~0.134)
Relationship Status (Ref: Single)	0.078** (0.050~0.212)	0.072** (0.039~0.202)	0.072** (0.039~0.203)
Religious Belief (Ref: No)	−0.019 (−0.204~0.086)	−0.018 (−0.199~0.089)	−0.019 (−0.202~0.086)
Employment Status (Ref: No)	−0.017 (−0.099 ~ 0.49)	−0.025 (−0.110 ~ 0.36)	−0.027 (−0.113 ~ 0.34)
City Living Experience (Ref: No)	0.037 (−0.019~0.136)	0.027 (−0.037~0.123)	0.027 (−0.037~0.123)
Communist Party Membership (Ref: No)	0.115*** (0.138~0.336)	0.102*** (0.107~0.314)	0.104*** (0.109~0.317)
Age	−0.054* (−0.005~0.000)	−0.048 (−0.005~0.000)	−0.049 (−0.005~0.000)
Annual Household Income	0.075** (0.019~0.094)	0.068** (0.014~0.089)	0.069** (0.014~0.089)
Main predictors			
Biospheric values (BV)		0.111*** (0.056~0.144)	0.079* (0.017~0.126)
Environmental risk perception (ERP)		−0.045 (−0.059~0.002)	−0.029 (−0.056~0.020)
Knowledge of environmental policies (KEP)		0.095*** (0.070~0.216)	0.089* (0.031~0.237)
Educational groups (Ref: Less-educated)			
Moderately educated (ME)		0.000 (−0.088~0.088)	−0.008 (−0.103~0.077)
Highly educated (HE)		−0.047 (−0.224~0.027)	−0.033 (−0.221~0.083)
Interaction terms (BV × Education)			
BV × ME			0.051 (−0.011~0.194)
BV × HE			0.019 (−0.097~0.199)
Interaction terms (ERP × Education)			
ERP × ME			−0.017 (−0.090~0.049)
ERP × HE			−0.029 (−0.156~0.052)
Interaction terms (KEP × Education)			
KEP × ME			0.012 (−0.130~0.198)
KEP × HE			−0.008 (−0.220~0.170)
Model statistics			
F	8.296***	8.289***	5.894***
R²	0.037	0.059	0.061
ΔR²	0.037	0.022	0.002

p*<0.05; p**<0.01; p***<0.001

In Model 1, being male (β = 0.055, *P* = .024), being in a relationship (β = 0.078, *P* = .002), being a member of the Communist Party (β = 0.115, *P* < .001), younger age (β = − 0.054, *P* = .047), and having a higher annual household income (β = 0.075, *P* = .003) were significantly associated with pro-environmental behavioral intentions. In Model 2, biospheric values (β = 0.111, *P* < .001) and knowledge of environmental policies (β = 0.095, *P* < .001) emerged as significant positive predictors, while environmental risk perception was not significant (β = − 0.045, *P* = .068). Neither the moderately educated group (β = 0.000, *P* = .998) nor the highly educated group (β = − 0.047, *P* = .125) differed significantly from the less-educated reference group in terms of their behavioral intentions. In Model 3, none of the interaction terms reached statistical significance. Although the interaction analysis does not provide formal statistical evidence for differential relationships across educational attainment, this discrepancy between the subgroup analysis and the interaction analysis may be attributable to the relatively small size of each group and the resulting loss of statistical power in detecting interaction effects.

## Discussion

This study contributes to environmental psychology by suggesting that the psychological pathways to access pro-environmental behavioral intentions are not consistent but are systematically differentiated by educational levels. We will discuss the results of this paper.

### Main findings

The statistical analysis yielded several key findings regarding the proposed hypotheses: First, Hypothesis 1, which hypothesized that biospheric values are positively associated with pro-environmental behavioral intentions, was partially supported. A significant positive association was found only in the less-educated and moderately educated groups. This suggests that biospheric values show a significant association with behavioral intentions mainly among individuals with lower to moderate education levels.

This result partially supports the value-belief-norm (VBN) theory, which proposes that biospheric values activate personal norms for pro-environmental actions. However, the theory does not claim that this effect does not exist among highly educated citizens. Therefore, these results indicate a limited condition of the VBN theory. Instead, the link between values and intentions may weaken when individuals have enough cognitive resources to depend on knowledge - based mechanisms.

Second, Hypothesis 2, which hypothesized that environmental risk perception is positively associated with pro-environmental behavioral intentions, was not supported. Across all three educational groups, environmental risk perception showed no significant effect on behavioral intentions. This null finding suggests that, in the Chinese context, perceiving environmental pollution does not necessarily lead to stronger pro-environmental intentions. We employ multiple theoretical perspectives to interpret this outcome.

From the perspective of perceived behavioral control, a core component of the Theory of Planned Behavior, some individuals may recognize environmental problems but feel unable to alleviate them through personal actions. For example, air or water pollution is often caused by industrial activities that are beyond individual control [58]. When people believe that their own efforts make little difference, risk perception fails to motivate actions.

In addition, according to attribution theory, some individuals may attribute the responsibility for pollution prevention and management to external actors, such as governments, rather than to themselves [59]. In China’s government-led environmental governance system, citizens may be accustomed to top-down policy implementation and regard environmental protection as the responsibility shouldered by governments [10]. Consequently, due to the perceived locus of responsibility lying outside themselves, personal risk perception may not be translated into concrete actions.

Briefly, we do not claim that pollution perception is unimportant. Instead, its influence may be conditional on perceived efficacy, attribution of responsibility, or other factors. Future studies may empirically test these mechanisms. This result challenges the risk perception theory, which predicts a positive effect of perceived risks on behavioral intentions. This finding reveals that in contexts where environmental governance is perceived as government-led, the association between risk perceptions and actions is not automatic but depends on efficacy beliefs and the attribution of responsibility. This result undoubtedly extends the risk perception theory by specifying it in non-Western and government-led contexts.

Third, Hypothesis 3, which hypothesized that knowledge of environmental policies is positively associated with pro-environmental behavioral intentions, received partial support. Knowledge of environmental policies was only significantly positively associated with behavioral intentions in the less- and moderately educated groups. This reveals that having knowledge of environmental policies plays a more important role in shaping citizens’ intentions in the less- and moderately educated groups, but not in the highly educated group. For individuals with low to moderate educational attainment, relevant knowledge may provide necessary cognitive guidance for pro-environmental actions. In contrast, those who are highly educated may already possess alternative cognitive resources, so relevant policy knowledge may not be crucial in driving intentions. This nuanced result supports the Theory of Planned Behavior’s proposition regarding perceived behavioral control [60]. Knowledge enhances perceived control, yet this effect becomes less pronounced when individuals already possess sufficient cognitive capacity.

Fourth, Hypothesis 4 received partial support from the subgroup analysis. The results showed the associations of biospheric values and knowledge of environmental policies with pro-environmental behavioral intentions were significant only for the less- and moderately educated groups, but not for the highly educated group. However, the interaction test did not yield significant interaction term, suggesting that the observed differences across groups should be interpreted with caution. This pattern of differences across groups is consistent with Inglehart’s post-materialism theory, which posits that educational expansion changes value priorities [61]. Our findings confirm that education not only promotes pro-environmental values but also differentiates which psychological pathways are more relevant for forming intentions.

For less- and moderately educated individuals, both biospheric values and knowledge of environmental policies are significant drivers. However, they did not reach statistical significance for the highly educated group. This result suggests that other unmeasured factors may be crucial for shaping their pro-environmental intentions.

The non-significant results for the highly educated group require further exploration. One possible explanation is a ceiling effect. As shown in Table 3, the highly educated group already reported the highest levels of pro-environmental intentions (M = 4.02). With the mean so close to the maximum possible score of 5, the share of variance left to explain is restricted, making it statistically challenging for predictors to yield significant associations. Moreover, the factors shaping intentions in this group may be more diverse and complex, extending beyond the simple motivational or cognitive drivers examined in this study. For highly educated individuals, who possess ample cognitive resources, their pro-environmental intentions might be more strongly influenced by a confluence of factors: professional norms, social network influences, a nuanced understanding of systemic environmental issues, and a strong sense of personal efficacy and responsibility [43]. For them, ecological values and basic policy knowledge may operate as necessary background conditions rather than as direct, proximal drivers of intention.

According to the results of empirical analysis, several important findings are worthy of discussion. First, there were significant differences across educational groups in terms of the levels of biospheric values, environmental risk perception, knowledge of environmental policies, and pro-environmental intentions. Regarding biospheric values and pro-environmental intentions, both the moderately and highly educated groups reported significantly higher levels than the less-educated group, while there was no significant difference between the two higher-educated groups. This indicates a threshold effect, where attaining at least a senior high school level of education is associated with an evident increase in environmental concerns and intended actions.

Second, environmental risk perception and knowledge of environmental policies showed an evident gradual increase with educational attainment. Regarding the reason why citizens with a higher educational level perceive more serious pollution, education is associated with higher awareness of environmental issues and access to information released by the government [62]. Moreover, those with a higher educational level may assess environmental quality using stricter standards [63]. However, according to the regression results, although higher education is correlated with a stronger perception of pollution, a stronger perception of pollution does not correspond to stronger intentions to engage in environmental protection. One possible interpretation is that there is a gap between cognitive awareness and the motivational process, a pattern referred to as “cognitive self-interest” [64]. However, other explanations should not be neglected. The null effect may result from measurement issues or from political and cultural factors. For example, individuals’ perception of pollution severity may not reflect the individual threats they face. In addition, individuals may rely more on governments than on personal actions. A more cautious conclusion is that risk perception alone does not absolutely drive intentions unless accompanied by perceived efficacy or action-related knowledge. Future research should empirically test these competing mechanisms.

Third, regarding the role of demographic controls across educational level groups, for example, being male was significantly associated with stronger intentions only in the less-educated group. Some previous studies have indicated that women exhibit higher levels of pro-environmental behavior than men [65, 66]. The result of this study seems contradictory to previous studies. In the less-educated group, traditional gender roles might be stronger, and some men may think that they should set an example for their families, thereby having a stronger sense of action regarding environmental protection [67]. In addition, they may publicly demonstrate their environmental awareness based on “face-saving” considerations [68]. On the contrary, higher-educated individuals may hold stronger gender equality attitudes, and then the gender difference in behavioral intentions is weakened [69].

Concerning Communist Party membership, it was significantly associated with stronger pro-environmental behavioral intentions among the less- and moderately educated groups, but not among the highly educated groups. This reflects the role of government-led environmental activities in shaping pro-environmental norms among party members with relatively lower educational attainment. On the contrary, for highly educated individuals, their environmental engagement is already supported by their psychological resources, so party affiliation appears less relevant.

Regarding the finding that higher household income was significantly associated with stronger behavioral intentions only in the less- and moderately-educated groups, economic factors may be less of a motivator or barrier for those with higher degrees. Generally speaking, those who are highly educated can easily engage in well-paying jobs [70]. Their environmental engagement may stem from personal values or social responsibility rather than economic considerations. In regard to the finding that relationship status was a significant variable only for the moderately and highly educated groups, among those with moderate to high educational attainment, being in a relationship may often entail frequent shared discussions on social and public issues [71]. Through peer encouragement, they may co-occur with stronger behavioral intentions to engage in environmental protection in their daily lives. Conversely, for less-educated individuals in a relationship, couples may communicate and cooperate regarding basic needs rather than public affairs such as environmental protection [72].

Another interesting finding is that, among the highly educated, although the level of biospheric values was the same as that of the moderately educated, this factor was not significantly associated with their behavioral intentions. For highly educated citizens, their intentions may show a stronger association with other factors such as systematic knowledge or practical considerations rather than moral values alone. On the contrary, for those with lower and moderate education levels, biospheric values shows a stronger association with intentions.

This may reflect that when individuals have difficulties obtaining or understanding complicated policy information, value-based appeals and moral demands may be more strongly associated with environmental engagement. On the other hand, the role of knowledge of environmental policies in the less- and moderately educated groups reveals that understanding concrete policies and action plans is associated with citizens forming behavioral intentions, especially among those with lower to moderate educational attainment.

Overall, the results of this study reveal a nuanced phenomenon. Although higher education is associated with greater environmental knowledge, awareness, and engagement intentions, the associations with these intentions are not consistent. Biospheric values and knowledge of environmental policies show a stronger association for those with lower to moderate educational attainment, even though their intentions might be lower. This finding implies that recognizing environmental problems does not absolutely translate into actions, possibly due to insufficient perceived behavioral control or low response efficacy. In addition, individuals may defer responsibility rather than act personally in contexts where environmental governance is principally government-led. Therefore, multiple mechanisms may explain the null effect. These findings prove that educational attainment is not only correlated with pro-environmental intentions but also a key factor that differentiates the patterns of those intentions.

Theoretically, these findings extend the post-materialism framework by specifying the differential psychological mechanisms across educational groups. The nuanced pattern indicates that education not only enhances environmental values but also shifts the basis of environmental engagement from moral motivation to cognitive competence. Thus, China, with its educational expansion and government-led environmental governance, offers an important context for observing how education and modernization influence the psychological bases of pro-environmental behavior.

However, it is important to interpret these subgroup differences with caution. The formal interaction analysis did not yield statistically significant interaction terms, indicating that the moderating effect of education was not robustly confirmed across the full sample. This discrepancy between the subgroup analysis and the interaction test is likely attributable to methodological factors. Stratifying the sample into three educational groups substantially reduced the statistical power of each subgroup analysis, making it more difficult to detect significant within-group effects [73]. In contrast, while the pooled interaction analysis offers greater statistical power, it may also obscure nuanced, non-linear moderation patterns that are specific to certain subgroups. Therefore, while the subgroup analyses offer suggestive evidence of differential patterns, the findings should be regarded as exploratory and hypothesis-generating, and require confirmation in future studies using larger, more balanced samples across educational strata.

### Limitations

This study acknowledges the presence of certain limitations that could be addressed in future research endeavors. One limitation of this study is the use of secondary data. Although this approach provided convenient access to the data, it also imposed certain restrictions. It is worth noting that the questionnaire used in this study, the Chinese General Social Survey, was meticulously designed and proficiently administered by academic agencies. This allowed researchers to directly access the data. However, it was not possible to customize the questions to meet our specific requirements. We had to consider how well the existing items in the questionnaire could address the research inquiries. In subsequent studies, scholars may consider revising and enhancing a more targeted questionnaire related to this subject matter. Second, the cross-sectional design limits the ability to infer causal relationships between the independent variables and behavioral intentions. If the Chinese General Social Survey conducts surveys with the same items in the future, longitudinal studies could establish causal pathways. Third, the self-reported measures may introduce social desirability bias for socially valued outcomes [74]. Researchers may incorporate experimental designs to supplement self-report data. Finally, beyond educational level, other potentially strong predictors may merit investigation in future research. These include: perceived behavioral control and subjective norms, which are core components of the Theory of Planned Behavior that were not directly measured in our study; environmental self-efficacy, which reflects individuals’ confidence in their ability to carry out effective environmental actions; trust in government and institutional effectiveness, which may shape citizens’ willingness to engage in pro-environmental behaviors within a government-led governance system; and media consumption habits, which influence exposure to environmental information and may moderate the translation of environmental knowledge into behavioral intentions. Incorporating these variables into future studies will help develop more comprehensive models with greater explanatory power, enabling a clearer understanding of the differentiated psychological pathways across educational strata.

Furthermore, the explanatory power of our regression models, as reflected by the R^2^ values, remained relatively modest across all educational groups. This indicates that the three psychological predictors in this study account for only a limited portion of the variance in pro-environmental behavioral intentions. It means that other potentially powerful predictors are not included in our models. On the one hand, this may be a limitation. On the other hand, this may be an opportunity for future research to develop more comprehensive models that incorporate other determinants of pro-environmental intentions. In addition, longitudinal designs or experimental methods may help achieve greater explanatory power by capturing changes in intentions and behaviors over time.

### Practical implications

The findings of this study offer some valuable recommendations for promoting the pro-environmental engagement of Chinese citizens. Appropriate measures should be customized to reflect the specific cognitive conditions of groups with different educational levels. First, for individuals with low to moderate educational attainment, it is important to strengthen their ecocentric values in daily life. Since the readers may not have a very high level of education, it is better to use simple and easy-to-understand words to express the precious value of nature, the importance of the ecological environment to people, and what people can do for the environment, rather than using difficult professional expressions. In addition, with the rapid and prosperous development of short videos in recent years, governmental sectors and social organizations related with educational and environmental affairs could make attractive and interesting short videos to support citizens’ moral and affective connection with the environment.

Second, the finding that there is a positive association between knowledge of environmental policies and pro-environmental behavioral intentions among the less- and moderately educated groups has practical implications. Although the association did not reach statistical significance for the highly educated group, the positive trend suggests that policy knowledge may still be relevant for this population. Our measure specifically captures familiarity with national environmental policies. Policy makers and educators may prioritize the dissemination of policy-related knowledge. The dissemination of knowledge should not be limited to schools, and correct, practical, and up-to-date information related to environmental protection should be transmitted through accessible channels. For example, both public and private organizations can use employee meetings to share and explain the latest key national policies related to environmental protection. To avoid employee indifference or resistance, policy messages should be delivered in a less boring and didactic way. Clear, brief, lively, and diverse methods, such as appropriate online media platforms, can be skillfully used to provide clear and accessible information. Similarly, communities can organize various activities for residents to strengthen community bonds and promote environmental policies.

Third, despite the null association between environmental risk perception and behavioral intentions, it cannot be concluded that risk communication is unhelpful. Our findings suggest that merely emphasizing pollution harms is unlikely to be sufficient. Communication strategies should provide risk information along with executable knowledge and messages that enhance efficacy.

## Conclusions

This study unveils the interplay among biospheric values, environmental risk perception, knowledge of environmental policies, and educational attainment in association with the pro-environmental behavioral intentions of Chinese citizens. The results show that biospheric values and knowledge of environmental policies show a stronger association among individuals with lower and moderate educational levels, while environmental risk perception is not significantly associated with behavioral intentions in any group.

These findings bring important implications for environmental policy and education. Stakeholders related to the environment and education should emphasize improving knowledge of environmental policies through adequate communication strategies, especially paying attention to those with lower educational levels. For these groups, cultivating ecocentric values through narratives close to daily life may show a stronger association with behavioral intentions than monotonous propaganda alone. Moreover, emphasizing pollution threats in the living environment may be less closely related to citizens’ environmental engagement.

This study highlights the comparative education approaches in environmental governance. By customizing strategies for different educational groups, relevant departments and personnel can support the behavioral intentions to engage in environmental protection.

This study also makes a theoretical contribution by demonstrating that the relative importance of different psychological pathways vary across educational groups. By examining these relationships in China, this study provides boundary conditions for existing theories developed in Western contexts. The findings help researchers pay attention to institutional and educational contexts that shape the psychological mechanisms of environmental engagement.

## Data Availability

The datasets used and/or analyzed during the current study are available from http://cgss.ruc.edu.cn/ upon reasonable request.
